# Surgery v. Medicine

**Published:** 1865-12

**Authors:** 


### SURGERY *v.* MEDICINE.

After an experience of fifty years, says Sir Benjamin Brodie in his *Autobiography*, I am confirmed in the opinion that the pursuit of what is called pure surgery, such as it is in large cities, in connection with a hospital and a medical school, is more replete with interest, and, on the whole, more satisfactory, than any of the other branches into which the *ars mendendi* is divided.—*Surg. and Med. Reporter.*

